# Synthesis and In Vitro Activity Assessment of Novel Silicon Oxycarbide-Based Bioactive Glasses

**DOI:** 10.3390/ma9120959

**Published:** 2016-11-24

**Authors:** Isabel Gonzalo-Juan, Rainer Detsch, Sanjay Mathur, Emanuel Ionescu, Aldo R. Boccaccini, Ralf Riedel

**Affiliations:** 1 FB Material-und Geowissenschaften, Technische Universität Darmstadt, Jovanka-Bontschits-Strasse 2, Darmstadt D-64287, Germany; ionescu@materials.tu-darmstadt.de (E.I.); riedel@materials.tu-darmstadt.de (R.R.); 2 Department of Materials Science and Engineering, Institute of Biomaterials, University of Erlangen-Nuremberg, Cauerstrasse 6, Erlangen D-91058, Germany; rainer.detsch@fau.de; 3 Institute for Inorganic Chemistry, University of Cologne, Greinstrasse 6, Köln D-50939, Germany; sanjay.mathur@uni-koeln.de

**Keywords:** SiOC, Ca- and Mg-modified silicon oxycarbide, polymeric single source precursors, bioactive glass, bioactivity, cytotoxicity

## Abstract

Novel bioactive glasses based on a Ca- and Mg-modified silicon oxycarbide (SiCaMgOC) were prepared from a polymeric single-source precursor, and their in vitro activity towards hydroxyapatite mineralization was investigated upon incubating the samples in simulated body fluid (SBF) at 37 °C. The as-prepared materials exhibit an outstanding resistance against devitrification processes and maintain their amorphous nature even after exposure to 1300 °C. The X-ray diffraction (XRD) analysis of the SiCaMgOC samples after the SBF test showed characteristic reflections of apatite after only three days, indicating a promising bioactivity. The release kinetics of the Ca^2+^ and Mg^2+^ and the adsorption of H^+^ after immersion of SiCaMgOC in simulated body fluid for different soaking times were analyzed via optical emission spectroscopy. The results show that the mechanism of formation of apatite on the surface of the SiCaMgOC powders is similar to that observed for standard (silicate) bioactive glasses. A preliminary cytotoxicity investigation of the SiOC-based bioactive glasses was performed in the presence of mouse embryonic fibroblasts (MEF) as well as human embryonic kidney cells (HEK-293). Due to their excellent high-temperature crystallization resistance in addition to bioactivity, the Ca- and Mg-modified SiOC glasses presented here might have high potential in applications related to bone repair and regeneration.

## 1. Introduction

Bioactive glasses (BGs) and bioactive glass–ceramics (BGCs) of specific compositions have been studied for the last four decades as suitable materials for bone regeneration purposes since Hench et al. found that the melt-quenched CaO–SiO_2_–P_2_O_5_–Na_2_O system (e.g., Bioglass^®^ 45S5) was able to tightly bond to the bone [1]. The bioactivity of BGs and BGCs is attributed to the formation of a bone-like hydroxyl-carbonated apatite (HCA) layer on their surface in (simulated) biological environments, which thus induces a strong bonding of the BG/BGC surface with bone tissues. It is also well established that silicon-based biomaterials play an essential role in bone formation, as the presence of silicon in bioactive ceramics and glasses has a significant effect in the osteogenesis process [2]. Several BGs containing SiO_2_ as network former and glass–ceramic compositions are able to stimulate tissue regeneration by inducing the formation of surface active layers much faster than other bioactive ceramics that do not contain Si in their composition; for example, silica-based bioactive glasses induce the formation of a surface-active layer three times faster than does hydroxyapatite [3,4,5,6].

Porous bioactive silicate glass scaffolds are commonly prepared by heating (sintering) the glass particles that have already formed into the desired 3-D geometry [7]. In the case of 45S5 Bioglass^®^, because of the limited ability of the glass to sinter by viscous flow above its glass transition temperature (*T_g_*), and the narrow temperature window between *T_g_* and the onset of crystallization, difficulties are encountered while trying to sinter the particles into a dense amorphous network [8]. Usually, the glass devitrifies during sintering to form a predominantly crystalline phase (Na_2_O−2CaO−3SiO_2_). The co-existence of a crystalline phase and a residual glassy phase affects the mechanical properties of scaffolds; consequently, the processed 45S5 Bioglass^®^ scaffolds often have low strength [8]. In order to overcome this drawback and to increase the crystallization temperature of BGs, significant efforts have been made with a special focus on developing novel compositions using new synthesis methods such as the sol–gel process [9,10,11,12]. An early extensive investigation of new glass compositions was conducted by Brink and colleagues to identify alternative bioactive glass compositions suitable for use as processed materials such as coatings and scaffolds [13,14]. The latter investigators developed 26 different glasses within the Na_2_O–K_2_O–MgO–CaO–B_2_O_3_–P_2_O_5_–SiO_2_ system and implanted samples of each glass in rabbit tibia for 8 weeks to test their bioactivity in vivo. Follow-up physical analyses of the resected specimens revealed that 10 of the 26 compositions showed direct bonding to bone via a bioactive calcium phosphate layer. Among them, only a silicate bioactive glass, designated 13–93 [13,14], shows better processing characteristics by viscous flow sintering (larger processing window between *T_g_* and the onset of crystallization), but it degrades (and converts to an HCA-like material) more slowly [15], when compared to 45S5 BG.

In the present work, we report for the first time on the preparation of novel bioactive glass compositions based on silicon oxycarbide, which are able to keep their amorphous nature up to temperatures as high as 1300 °C. These materials are intended for bone regeneration applications; therefore, the degree of their in vitro activity towards HCA mineralization was investigated after incubating the samples in simulated body fluid (SBF) at 37 °C. The formation of HCA on the surface of the glass was assessed by X-ray diffraction (XRD) analysis, while the kinetics of its biodegradation (biomineralization) was studied mainly by optical emission spectroscopy measurements. In addition to bioactivity assessment, the possible cytotoxicity of the new glasses in the presence of various cells was investigated.

## 2. Materials and Methods

### 2.1. SiOC and SiCaMgOC Preparation

The single-source precursor for SiCaMgOC was prepared upon reacting a solution of 5 g of a polysilsesquioxane (MK Belsil PMS, Wacker, Burghausen, Germany) in 30 mL of methanol with a methanolic solution containing 1.25 g of magnesium acetylacetonate (Mg(acac)_2_, Sigma Aldrich, Darmstadt, Germany) and 0.9 g of calcium acetylacetonate (Ca(acac)_2_, Sigma Aldrich, Darmstadt, Germany). After mixing PMS with the corresponding metal precursors, the reaction solution was stirred overnight at room temperature. Subsequently, the solvent was removed under vacuum (10^−2^ mbar). The obtained single-source precursor was cross-linked at 250 °C and converted upon pyrolysis at 1100 °C and at 1300 °C during 3 h in argon to SiCaMgOC. The mass loss occurring during the conversion of the Mg- and Ca-modified polysilsesquioxane into SiMgCaOC glass was ca. 31 wt % and typically correlates with the removal of the organic groups of the polymeric precursor as gaseous species, e.g., hydrocarbons and hydrogen.

### 2.2. Structural Characterization of SiOC and SiCaMgOC Powders

Fourier transformed infrared (FTIR) spectra were collected using a Bruker Vertex 70 FT-IR instrument (Bruker, Madison, WI, USA) in attenuated total reflectance geometry (ATR). X-ray diffraction (XRD) measurements were performed with a STOE X-ray diffractometer (Stoe & Cie GmbH, Darmstadt, Germany) in transmission geometry (Mo K_α_ radiation). The specific surface area (SSA) of the SiOC samples was measured by determining the N_2_-gas adsorption–desorption isotherms (Autosorb AS6, QuantaChrome, Odelzhausen, Germany). Specific surface areas were calculated by fitting the first seven points (relative pressures of 0.05–0.3) of the adsorption branch of the isotherm to the Brunauer–Emmett–Teller (BET) equation. The carbon content of the samples was determined with a carbon analyzer, CS 800 (Eltra GmbH, Neuss, Germany) and the oxygen content with a N/O analyzer, Leco TC-436 (Leco Corporation, St. Joseph, MI, USA) whereas silicon, calcium, and magnesium contents were determined at Mikroanalytisches Labor Pascher (Remagen, Germany) via chemical digestion followed by inductively coupled plasma optical emission spectroscopy (ICP-OES).

### 2.3. In Vitro Acellular Assessment of the Bioactivity of the SiOC and SiCaMgOC Powders: Cytotoxicity Tests

Simulated body fluid (SBF) was prepared according to Kokubo’s method [16]. To produce 1 L of SBF, 700 mL of deionized water (DI) was placed in a 1 L polypropylene beaker and set at 37 ± 1.0 °C in a water bath. The solution was continuously stirred throughout. The reagents, provided by Sigma-Aldrich UK and used as received without further purification, were slowly added to the DI water. The pH was adjusted to 7.4 before use at 37 °C. Bioactive glass powders were immersed in SBF using a ratio of 75 mg of glass to 50 mL of SBF in an airtight polyethylene container. Samples were stirred and placed in a water bath at 37 °C and soaked for different durations. At the end of each time period, the sample was removed and the solids were collected. The powder was immediately washed with DI water and acetone. The filtered solution was collected to determine the ion concentrations using optical emission spectroscopy (Agilent 720 ICP-OES, Victoria, Australia). The pH of the solution was also measured.

Two cell biology tests were carried out to assess the biological response of the new materials.

In order to assess the cell compatibility of the materials, a direct method was applied. Mouse embryonic fibroblasts (MEF) were cultured for 24 h in a Dulbecco’s Modified Eagle Medium (DMEM) containing 10% Foetal Bovine Serum (FBS) and 1% penicillin-Streptomycin, at 37 °C, in a humidified atmosphere (air with 5% CO_2_). In the direct method, MEF cells were seeded directly onto the samples and two control materials surfaces (glass slides and polystyrene—cell culture plate) for 24 h. Afterwards, for the cell viability quantification, water-soluble tetrasodium (WST8-Test from Sigma Aldrich^®^, Darmstadt, Germany) was used as recommended by the manufacturer. Qualitative evaluation was performed using a fluorescence microscope ZEWASS Scope A1, with a digital camera AxioCam Icc 1 S/N 28591065, an HXP120C Kübler fluorescence light source, and green Calcein markers, and images were analyzed using Software ZEN^®^ (blue edition, 2006–2011 Carl Zeiss micro-Imaging GmbH, Jena, Germany) before cell seeding samples were sterilized at 121 °C for 20 min in an autoclave (DX-23 Systec, Linden, Germany).

The cytotoxicity of the SiOC powder samples was tested via an MTT assay [17] in the presence of HEK 293 cells (derived from human embryonic kidney). MTT (i.e., 3-[4,5-dimethylthiazol-2-yl]-2,5-diphenyltetrazolium bromide), which is yellow in color, is absorbed by the mitochondria and enzymatically converted by succinic dehydrogenase into formazan (purple color). Thus, HEK 293 cells were initially seeded (150 μg cells/mL) and treated with the SiOC powder particles (solid loadings of 50–20 μg/mL) and incubated for 24 and 48 h at 37 °C, in an atmosphere supplemented with 5% CO_2_. Subsequently, MTT was added (5 mg/mL) and the solutions were further incubated for 1 h at 37 °C, 5% CO_2_, in order to convert the yellow MTT by the viable cells into purple formazan crystals. The results from the MTT assay are expressed as the means of the absorptions at 630 nm; thus, the measured absorbance is correlated with the number of viable cells, and the cell viability (in %) at different soaking times and solid loadings can be indicated.

Statistical analysis of the activity of MEF cells was accomplished via one-way analysis of variance (ANOVA) on the different samples after 24 h of incubation. Statistical analyses were accomplished using Origin software (Origin 8.5G; OriginLab Corporation, Northampton, MA, USA). The number of samples was *N* = 6. The significance level was set as * *p* < 0.05, ** *p* < 0.01, and *** *p* < 0.001. For the comparison of the mean values, the Tukey test was used.

## 3. Results and Discussion

### 3.1. Material Characterization and Acellular Bioactivity

**Figure 1:**
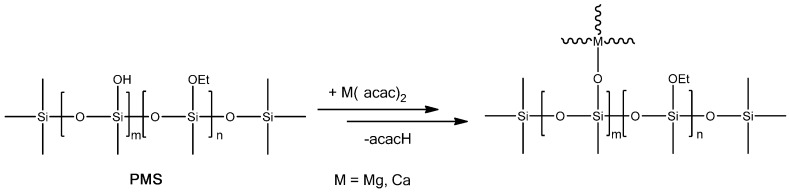
Sketch of the synthesis of the single-source precursor for SiCaMgOC upon chemical modification of PMS with acetyl acetonates of Ca and Mg.

In the present work, SiOC and SiCaMgOC were prepared starting from a polysilsesquioxane and a Ca- and Mg-modified polysilsesquioxane, respectively. The polysilsesquioxane MK Belsil PMS with the chemical composition Si_1_O_3_C_1_H_3.3_ is commercially available and contains cross-linkable hydroxy and ethoxy groups, which can further react with metal alkoxides, acetylacetonates, or acetates [18,19,20,21]. The average molecular weight of MK Belsil PMS has been indicated in the literature [22] to be ca. 10,000 g/mol; thus, the molar ratio Si:Ca:Mg in the single-source precursor was set to ca. 10:1:1. FTIR spectroscopy measurements (data not shown here) indicate that the reaction of the used metal precursors with PMS leads to the formation of Si–O–M units (see Figure 1), as revealed by the appearance of an absorption band at ~950 cm^−1^, and agree with previously reported results.

**Figure 2:**
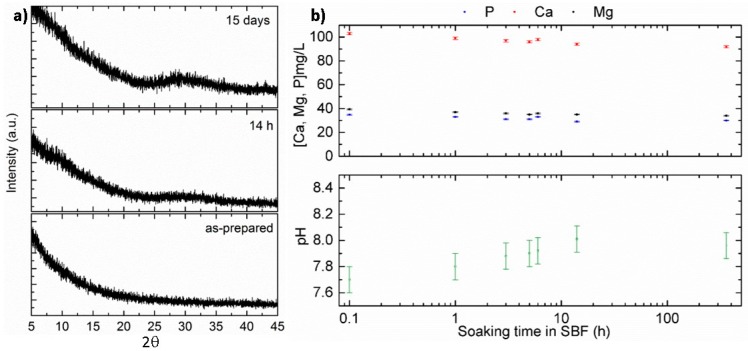
(**a**) XRD patterns of as-prepared SiOC powders (**bottom**) as well as after soaking in SBF [28] at 37 °C for 14 h (**middle**) and 15 days (**top**); (**b**) evolution of the content of Ca, Mg, and P in SBF (**above**) and pH (**below**) as a function of the soaking time during the SBF exposure of SiOC powders.

The as-prepared SiOC and SiCaMgOC samples were shown to be X-ray amorphous. They were investigated for the first time in vitro concerning their bioactivity related to the mineralization of hydroxyapatite upon SBF exposure. Figure 2a shows the XRD patterns of the as-prepared SiOC sample as well as after soaking in SBF for 14 h and 2 weeks. No diffraction peaks associated with the mineralization of hydroxyapatite (HCA) were detected upon incubating the SiOC sample in SBF. The concentrations of Ca, Mg, and P in the simulated body medium remained constant during the time the powders were exposed to SBF. The slight increase in the pH value from 7.7 to 8.0 (±0.1), which was detected during the incubation in SBF, may be a consequence of the formation of a layer of silica gel on the surface of the SiOC particles. Thus, the results obtained during the SBF test of SiOC indicate that the samples were not bioactive with respect to HCA mineralization.

In order to convert the SiOC particles into a bioactive material, the network of the glass was modified with Ca and Mg. Ca was chosen, as it is the main component of biological apatite and favors osteoblast proliferation, differentiation, and extracellular proliferation [23,24,25]. Besides Ca, Mg was also considered as a network modifier due to it is essential role in bone metabolism and its stimulating effects in the formation of new bone [26,27].

**Figure 3:**
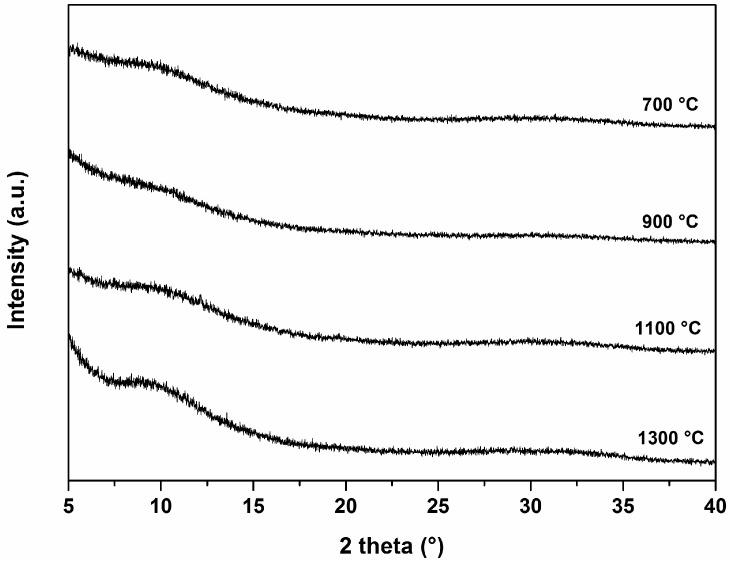
Diffraction patterns of SiCaMgOC glass pyrolyzed at 700, 900, 1100, and 1300 °C.

The chemical composition of the SiCaMgOC glass powder was determined to be Si_1_Mg_0.07_Ca_0.05_O_1.73_C_0.96_. Considering that the Ca and Mg is bonded to oxygen, the rest of the oxygen is bonded to silicon, the rest of the silicon is bonded to carbon, and the rest of the carbon is present as the secondary segregated phase, the molar fractions of CaO and MgO can be estimated from the elemental analysis data [19,29]. The molar fractions of MgO and CaO within the SiCaMgOC glass were 4.46 and 6.25 mol %, respectively, and thus significantly lower than the molar fraction of CaO in regular bioactive glasses (e.g., in 45S5 BG, the content of CaO is 26.9 mol %). Interestingly, the SiCaMgOC samples were shown to keep their glassy nature even after annealing at 1300 °C (see Figure 3). Moreover, the SiCaMgOC powder exhibited a relatively high specific surface area (i.e., 20 m^2^·g^−1^, compared with SSA values of glasses quenched from the melt, which range from 0.20 to 0.36 m^2^·g^−1^ [28]). This feature has been shown to be beneficial for the formation of hydroxyapatite, as a large surface area in BGs, silica gel, and glass–ceramics provides a high amount of silanol (Si–OH) terminations, which act as nucleation sites for HCA [30,31]. However, although both powders exhibit similar SSA, only SiCaMgOC shows activity concerning HCA mineralization. Consequently, we can conclude that, in our particular case, the specific surface area contribution is not significant in the bioactivity of our materials.

The zeta potential of the SiCaMgOC-1100 powders in SBF at pH 7.5 ± 0.1 is −29.4 ± 7.9 mV, indicating a good stability of the suspension, which is thus beneficial for HCA mineralization (as the SSA of the powder is not affected during the SBF test). The negative zeta potential of the SiCaMgOC suspension in SBF can be related to the composition of the glass and the surrounding environment and has been shown to be necessary for the nucleation of HCA on silica gel [32,33]. Moreover, it has important biological effects in vivo [34] and promotes the attachment and proliferation of cells [35,36].

**Figure 4:**
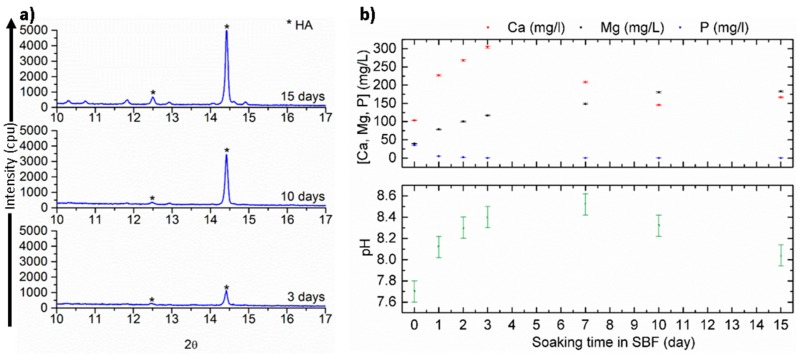
(**a**) XRD patterns of SiCaMgOC-1100 after soaking in SBF for 3, 10, and 15 days; (**b**) evolution of the Ca, Mg, and P contents in SBF (**above**) and pH (**below**) as a function of soaking time during the exposure of SiCaMgOC-1100 powder samples at 37 °C.

Figure 4a shows the XRD patterns of the SiCaMgOC glass prepared at 1100 °C (SiCaMgOC-1100 after SBF incubation for 3, 10, and 15 days. The characteristic XRD reflections of apatite are observed after three days of immersion in SBF. The main HCA reflections appearing at 12.4° and 14.4° (corresponding to the (002) and (211) diffraction planes, respectively) became sharper and more intense upon longer incubation times in SBF, suggesting a formation of apatite with higher crystallinity.

Figure 4b shows the evolution of the content of Ca, Mg, and P in the SBF after testing the SiCaMgOC-1100 powders for 1, 2, 3, 7, 10, and 15 days. The results show that the content of calcium and magnesium in SBF generally increased during the first three days of immersion in SBF, whereas phosphorous concentration decreased [28]. The pH of the SBF (Figure 4b) also increases with incubation time and accompanies the evolution of the contents of Ca and Mg ions in SBF. BG dissolution is due to the rapid ion exchange reactions between the glass network modifiers (Na^+^ and Ca^2+^) with protons (H^+^/H_3_O^+^) from the solution [37]. The pH of the SBF solution in the case of SiCaMgOC increases due to the ion exchange between Ca^2+^/Mg^2+^ (see the evolution of their concentration in SBF in Figure 4b) and H^+^. The concentration of Ca^2+^ decreases after five days of SBF exposure. This correlates to the depletion of phosphorus from the solution and occurs as a consequence of the crystallization of hydroxyapatite [37]. These results are consistent with the detection of crystalline apatite after three days of incubation by XRD. The pH value of the solution decreases after seven days of the SBF exposure of the SiCaMgOC glass and correlates to the crystallization of hydroxyapatite. This finding agrees with the observation of Cerruti et al., which indicates that the precipitation of calcium phosphates and carbonates during the SBF test are responsible for the shift of equilibria, such as HCO_3_^−^ ↔ CO_3_^2−^ + H^+^ and HPO_4_^2−^ ↔ PO_4_^3−^ + H^+^, to the product side and thus contribute to the decrease of the pH value of the solution upon extended exposure time [38].

### 3.2. Cytotoxicity Studies

SiOC-based monoliths and powders were studied with respect to their biocompatibility by performing two series of cytotoxicity tests. In a first experiment series, monolithic SiOC samples were tested in the presence of mouse embryonic fibroblast (MEF) cells. After 24 h of incubation, cell viability and cell morphology of MEF cells on the SiOC sample were assessed and compared with glass slides and polystyrene.

**Figure 5:**
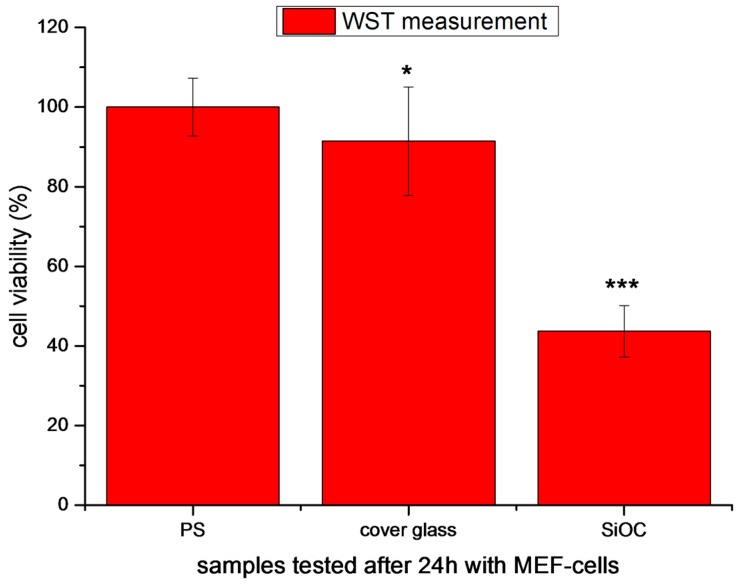
Cell viability measurement of MEF cells on polystyrene (PS), cover glass (CG), and SiOC samples after 24 h incubation. The significance level was set as * *p* < 0.05, ** *p* < 0.01, and *** *p* < 0.001. For the comparison of the mean values, the Tukey test was used.

The second experiment series involved the assessment of the cytotoxicity of SiOC powders in the presence of human embryonic kidney cells (HEK-293 cells). Whereas the first series of experiments represents a typical biocompatibility test of materials according to EN ISO 10993-5 biological evaluation of medical devices [39], the cytotoxicity test of the SiOC powder in combination with HEK-293 was performed in order to attain preliminary information about the effect of possible leaching species resulting from the exposure of SiOC to SBF (being either molecular species or small nanoparticles) or of debris (small particles) induced upon mechanical damage/erosion of monolithic SiOC-based implants on living cells and tissues. In Figure 5, the cellular viability of MEF cells in contact with the different samples (polystyrene (PS), cover glass (CG), and SiOC) after 24 h of incubation is shown. It can be observed that cell viability is significantly reduced in SiOC samples.

**Figure 6:**
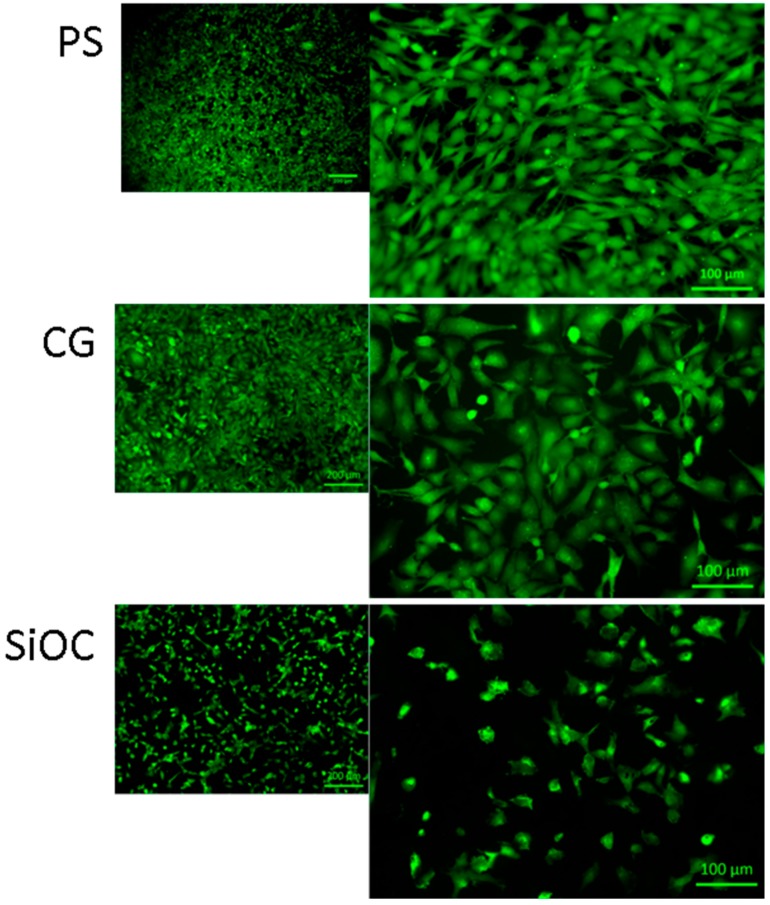
Fluorescence micrographs (FM) of MEF cells in direct contact with polystyrene (PS, **top**), cover glass (CG, **middle**), and SiOC (**bottom**) samples after 24 h of incubation. The green color corresponds to the staining of the cell cytosol of the living cells.

Figure 6 shows FM images of MEF cells in direct contact with the different samples after 24 h of incubation, whereby the green color corresponds to the staining of the cell cytosol of the living cells. In correlation to the viability measurements (Figure 5), there is a strong reduction in the cell layer, and the typical fibroblastic morphology is not expressed on SiOC samples. Although the attached cells on the surface, i.e., the living cells, are visualized in green color, there is no pronounced cell spreading on the SiOC surfaces.

SiOC-based powders with a specific surface area of ca. 20 m^2^/g were investigated with respect to their cytotoxicity against human embryonic kidney (HEK-293) cells in a MTT assay test. The viability of the cells was shown to be very high at low powder loadings (i.e., up to ca. 10 μg/mL), whereas a slight decrease in the viability was observed at higher solid loadings. There was no significant influence of the soaking time on the viability of the HEK-293 cells.

**Figure 7:**
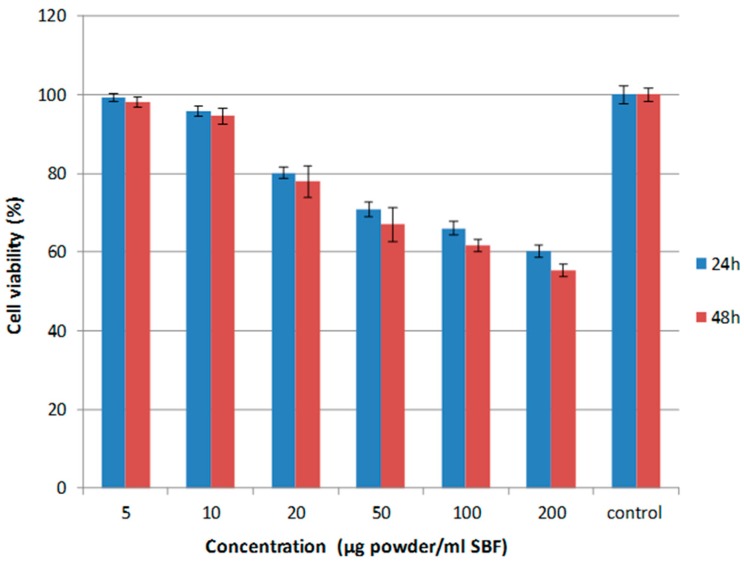
Cell viability of the SiCaMgOC-1100 powders with human embryonic kidney cells (HEK-293 cells).

It is known that polymer-derived glasses and glass–ceramics (and among them silicon oxycarbides) exhibit a secondary carbon phase within their microstructure, which is finely dispersed in the SiOC glassy matrix. The investigated sample contains for instance ca. 8 vol % segregated carbon [19,29]. In order to rationalize a possible effect of the secondary carbon phase on the cytotoxicity of SiOC, a silicon oxycarbide material with relatively high volume fraction of secondary segregated carbon was also assessed concerning its toxicity against HEK-293 cells. The second SiOC composition was prepared starting from a vinyl-containing polysiloxane (RD-212, Starfire Systems) and contains ca. 20 vol % segregated carbon. Interestingly, the cytotoxicity behavior was quite similar to that of the SiOC glass containing 8 vol % segregated carbon; thus, one can conclude that the segregated carbon in SiOC materials does not affect its cytotoxicity (Figure 7).

## 4. Conclusions

Novel glass compositions based on SiOC and SiOC modified with Ca and Mg intended for bone regeneration applications were prepared, and their in vitro activity was investigated after incubating the samples in simulated body fluid (SBF) at 37 °C. Cell biology tests were carried out to investigate possible cytotoxicity. The as-prepared glasses were amorphous after sintering at 1300 °C. The formation of HCA on the surface of the glass after incubation was assessed by XRD, while the biodegradation of its components was studied mainly via optical emission spectroscopy. The characterization of the as-prepared SiOC samples sintered at different temperatures revealed that SiOC is not able to develop a HCA layer after immersion in body fluids. However, the SiOC samples modified with Ca and Mg showed characteristic reflections of apatite after three days of immersion in SBF. The chemical species released from SiCaMgOC-1100 powders after immersion in SBF, (Ca^2+^, Mg^2+^ and H^+^) for 1 day, 2 days, 3 days, 7 days, 10 days, and 2 weeks were analyzed via optical emission spectroscopy. These results show that the mechanism by which crystalline apatite particles are developed on the surface of SiOC modified with Ca and Mg powders is similar to the mechanism observed for other bioactive glasses.

The direct cell seeding method showed that SiOC surfaces inhibit the adhesion of MEF cells after 24 h of incubation. Moreover, this results in a reduction of cell viability. As these tests are the first investigations with such a material, further studies will be carried out to comprehensively assess the material cytotoxicity. In future, cell adhesion complexes and cell material interactions will be analyzed in detail. With this information, the materials will be further optimized to increase cell attachment and tissue development.
